# RIPK3 expression in cervical cancer cells is required for PolyIC-induced necroptosis, IL-1α release, and efficient paracrine dendritic cell activation

**DOI:** 10.18632/oncotarget.3249

**Published:** 2015-03-20

**Authors:** Susanne V. Schmidt, Stefanie Seibert, Barbara Walch-Rückheim, Benjamin Vicinus, Eva-Maria Kamionka, Jennifer Pahne-Zeppenfeld, Erich-Franz Solomayer, Yoo-Jin Kim, Rainer M. Bohle, Sigrun Smola

**Affiliations:** ^1^ Center for Molecular Medicine Cologne and Institute of Virology, University of Cologne, Germany; ^2^ Institute of Virology, Saarland University, Homburg/Saar, Germany; ^3^ Department of Gynecology and Obstetrics, Saarland University, Homburg/Saar, Germany; ^4^ Department of Pathology, Saarland University, Homburg/Saar, Germany

**Keywords:** cervical cancer, human papilloma virus, dendritic cells, RIPK3

## Abstract

Previous studies have shown that cervical cancer cells only release low levels of pro-inflammatory cytokines owing to infection with human papillomaviruses. This results in low immunogenicity of the cancer cells. The viral dsRNA analog PolyIC has been suggested as a promising adjuvant for cervical cancer immunotherapy. However, little is known about the molecular requirements resulting in successful immune activation. Here, we demonstrate that stimulation of cervical cancer cells with PolyIC induced necroptotic cell death, which was strictly dependent on the expression of the receptor-interacting protein kinase RIPK3. Necroptotic cancer cells released interleukin-1α (IL-1α), which was required for powerful activation of dendritic cells (DC) to produce IL-12, a cytokine critical for anti-tumor responses. Again both, IL-1α release and DC activation, were strictly dependent on RIPK3 expression in the tumor cells. Of note, our *in situ* analyses revealed heterogeneous RIPK3 expression patterns in cervical squamous cell carcinomas and adenocarcinomas. In summary, our study identified a novel RIPK3-dependent mechanism that explains how PolyIC-treatment of cervical cancer cells leads to potent DC activation. Our findings suggest that the RIPK3 expression status in cervical cancer cells might critically influence the outcome of PolyIC-based immunotherapeutic approaches and should therefore be assessed prior to immunotherapy.

## INTRODUCTION

Cervical cancer represents the third most common cause of cancer-related death among women worldwide. It develops from persistent infection of the cervical epithelium with high-risk human papillomavirus (HPV) through well-defined stages of cervical intraepithelial neoplasia (CIN1–3) [1, 2]. Even in developed countries, the prognosis for women with diagnosed invasive cervical cancer remains poor [3–5]. The suppression of apoptosis by high-risk HPV oncoproteins [6, 7] may contribute to the reduced response of cervical cancers to chemoradiation [8].

Adjuvant therapy strategies against cancer are currently being explored to enhance cellular immune responses. These include immunotherapy regimens based on dendritic cells (DC), the major source of interleukin (IL)-12, which is required for effective cellular anti-tumor immune responses [9–14]. DC stimulation with polyinosinic:polycytidylic acid (PolyIC), a synthetic analog of viral dsRNA, has been proposed as a particularly promising adjuvant therapy [15–18]. In different regimens, the dsRNA is applied directly to DC generated from the individual patient's immune cells [15]. Alternative approaches are systemic application or local delivery of dsRNA, which may cause effects in immune cells as well as in cancer and other non-immune cell types [19]. The cytosolic application of PolyIC was recently shown to elicit an inflammatory form of cell death in ovarian cancer cells associated with the release of type I interferons (IFNs) and inflammatory cytokines, with subsequent induction of myeloid cell maturation and activation of natural killer cells [20]. In addition, normal human keratinocytes (the host cells of HPV) are strongly induced to produce inflammatory mediators, including tumor necrosis factor α (TNFα) and type I IFNs, upon stimulation with extracellular PolyIC; this in turn potently enhances myeloid DC activation [21, 22].

As a consequence of high-risk HPV infection and transformation, inflammatory cytokine production (including type I IFNs, TNFα, IL-1β, and different chemokines) is suppressed in keratinocytes [23–25]. The HPV oncoproteins also inhibit inflammatory cytokine expression in response to strong inducers such as dsRNA [25–28]. The situation is even aggravated in cervical cancer, in which the neoplastic cells generally display very low expression of inflammatory cytokines and chemokines [23, 29, 30]. An important example is IL-1β expression, which gradually decreases during cervical carcinogenesis [25]. In contrast, the alarmin IL-1α is stored intracellularly in keratinocytes and is expressed throughout cervical carcinogenesis [31]. IL-6 [32], a paracrine acting, pro-tumorigenic, immunosuppressive cytokine, is even up-regulated in cervical cancer [32–34]. In addition, immunostimulatory cytokines are downregulated in the microenvironment of invasive cervical carcinoma. Most importantly, the crucial cytokine IL-12 is significantly suppressed, indicating locally reduced immunity [35–37]. Therefore, immunotherapy approaches that raise IL-12 levels are of high interest. In patients, cervical cancers and pre-cancerous lesions should be well accessible to the local delivery of drugs such as dsRNA, and systemic application might also be feasible. However, thus far it is unclear how dsRNA-stimulated cervical cancer cells affect DC activation.

Here, we demonstrate that cervical cancer cells stimulated with PolyIC can potently increase IL-12 production in DC. This study identified an unexpected role for receptor-interacting protein kinase 3 (RIPK3), a key regulator of necroptosis [38], as a critical determinant of the capacity of PolyIC-stimulated cervical cancer to enhance DC activation.

## RESULTS

### Stimulation of C4-I cervical cancer cells with PolylC leads to cell death and enhances DC activation

**Figure 1:**
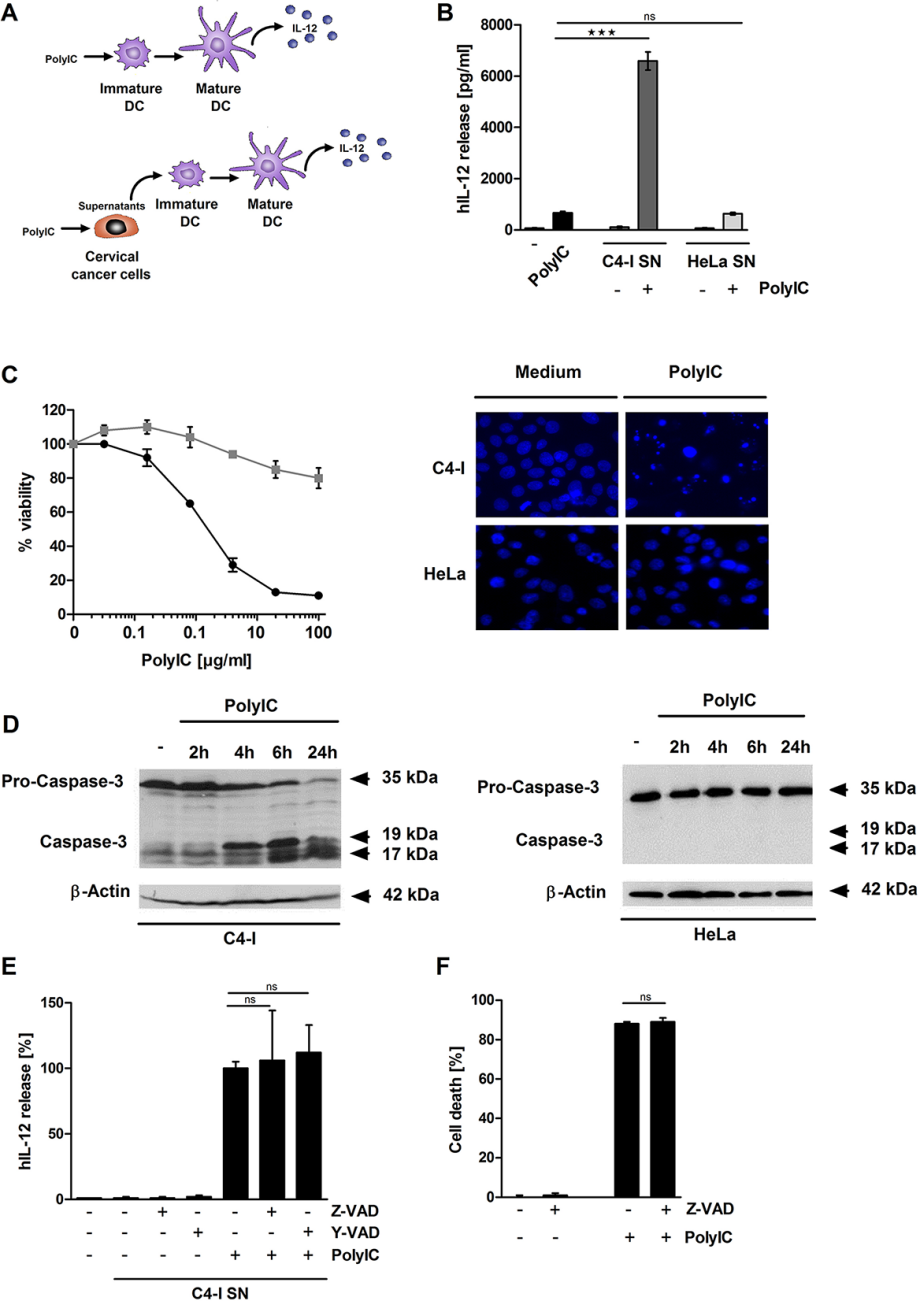
Supernatant from PolyIC-stimulated C4-I cells enhances the IL-12 production of DC **(A, B)** DC were incubated with medium, PolyIC, or supernatants from C4-I or HeLa cells treated with medium or PolyIC. The resulting supernatants from stimulated DC were analyzed for IL-12 expression by ELISA. The mean values ± SD from *n* = 2 experiments performed in duplicate are shown. PolyIC induces features of apoptotic cell death in C4-I cells. **(C)** C4-I cells (circles, black) and HeLa cells (squares, grey) were stimulated with serial dilutions of PolyIC for 24 h. Cell viability was assessed using the neutral red uptake method. The results from one experiment out of *n* = 3 experiments performed in duplicate are shown. C4-I and HeLa cells were stimulated as in (B) and stained with Hoechst (33342). Cells were analyzed using fluorescence microscopy at 200× magnification. **(D)** C4-I cells (left panels) and HeLa cells (right panel) were stimulated with PolyIC for 2, 4, 6, and 24 h. Whole cell extracts were analyzed for caspase-3 activation by Western blot. Equal loading was controlled using a β-actin-specific monoclonal antibody. Caspase inhibition affects neither the PolyIC-induced cell death nor the immunostimulatory potential of PolyIC-stimulated C4-I cells. **(E)** C4-I cells were incubated with Z-VAD or Y-VAD for 30 min and stimulated with PolyIC. Twenty-four hours later, supernatants were harvested and used for DC stimulation. The IL-12 expression induced by supernatants from PolyIC-treated C4-I cells was set at 100%. The mean values ± SD from *n* = 3 experiments performed in duplicate are shown. **(F)** C4-I cells were incubated with Z-VAD for 30 min, stimulated with PolyIC for 24 h, and assessed for cellular viability. Viability of medium-treated cells was set at 100%. The mean values ± SD from *n* = 2 experiments performed in duplicate are shown.

PolyIC has been suggested as a potential DC adjuvant against cancer, including cervical cancer [15]. We compared two different protocols for the stimulation of human DC. PolyIC was added directly to the DC (Figure 1A, upper panel) or applied to the cervical cancer cell lines C4-I and HeLa. Supernatants were harvested from the tumor cells 24 h later and used to stimulate DC (Figure 1A, lower panel). As an important indicator of DC activation, we measured the release of IL-12. This cytokine has a central role in the generation of anti-tumor immune responses. To our surprise, supernatant from PolyIC-stimulated C4-I cells led to a 10-fold higher production of IL-12p70 by DC, compared with DC directly activated with PolyIC (Figure 1B). This effect was not observed for supernatant from PolyIC-stimulated HeLa cells, which induced DC activation comparable to PolyIC applied directly. In contrast to normal human keratinocytes [22], PolyIC stimulation of C4-I cells elicited neither IFNβ nor TNFα secretion (data not shown). The lack of inflammatory cytokine production was in line with other studies performed with HPV-positive keratinocytes [34, 39].

In order to identify the molecular basis of the strong DC activation observed with PolyIC-stimulated C4-I but not HeLa cells, we explored the differences between the two cancer cell lines. The most obvious difference between C4-I and HeLa cells in response to PolyIC was cell death induction. While HeLa cells exhibited only a modest response (up to 20% cell death), PolyIC efficiently killed C4-I cells in a dose-dependent manner: maximum C4-I cell death neared 90% (Figure 1C, left panel). Strong chromatin condensation and fragmented nuclei revealed by Hoechst H33342 staining, as well as cell shrinkage (not shown), pointed to an apoptotic form of cell death (Figure 1C, right panel). Apoptotic cell death was further substantiated by the biochemical analysis of caspase-3 activation (Figure 1D). Four hours after the PolyIC stimulation of C4-I cells, a strong reduction of pro-caspase-3 (35 kDa) was detected, while the cleaved forms of caspase-3 (17 and 19 kDa) increased over time (Figure 1D, left panel). Again, this was in contrast to the behavior of PolyIC-stimulated HeLa cells (Figure 1D, right panel).

### Caspase inhibition neither blocks cell death nor suppresses the immunostimulatory potential of PolyIC-activated C4-I cells

To determine whether caspase-driven apoptosis was involved in the increased DC-stimulatory capacity of PolyIC-stimulated C4-I cells, they were pre-incubated with the pan-caspase inhibitor Z-VAD or the caspase-1 inhibitor Y-VAD as controls prior to PolyIC stimulation. Cell-free supernatants from C4-I were collected 24 h later and used in DC activation experiments. Again, supernatants from PolyIC-stimulated C4-I cells strongly induced DC to produce IL-12. However, neither the pan-caspase nor the caspase-1 inhibitor affected their DC-stimulatory capacity (Figure 1E). Notably, Z-VAD did not interfere with PolyIC-mediated cell death in C4-I cells (Figure 1F). The anti-apoptotic activity of Z-VAD was confirmed in a control experiment, in which it strongly inhibited TNFα/cycloheximide-induced cell death in HeLa cells (Supplementary Figure S1). These results demonstrated that PolyIC-induced caspase activation in C4-I cells did not contribute to their subsequent DC activation. Furthermore, these data indicated that PolyIC might also induce a form of non-apoptotic cell death in C4-I cells.

### PolyIC induces features of necrosis in C4-I cancer cells

**Figure 2:**
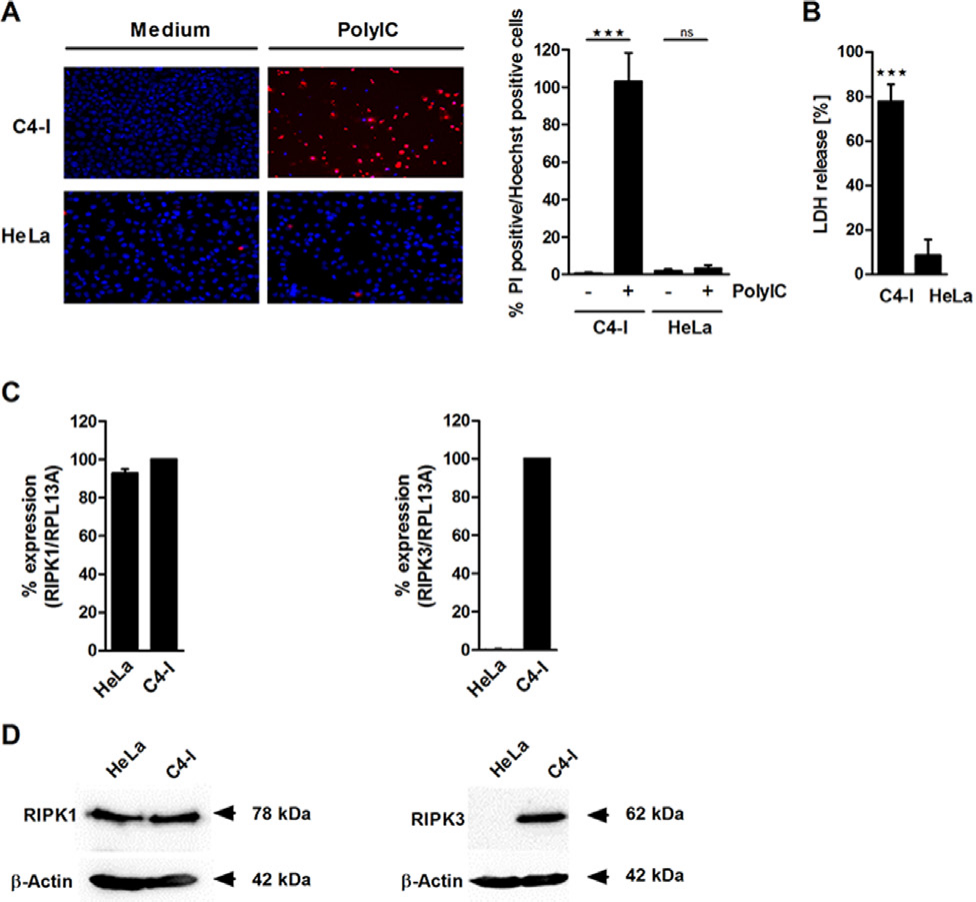
PolyIC induces features of necrosis in C4-I cells **(A)** C4-I and HeLa cells were stimulated with PolyIC for 24 h and stained with PI and Hoechst. Cells were analyzed using fluorescence microscopy at 200× magnification (left panel). Eight randomized pictures from *n* = 2 experiments were taken per condition and quantified for PI-positive cells (right panel). **(B)** C4-I and HeLa cells were stimulated as in (A) and analyzed for LDH release. The LDH release of the positive control (treatment with 2% Triton-X) was set at 100%. The results from *n* = 3 experiments performed in triplicate are shown. RIPK3 (but not RIPK1) is differentially expressed in C4-I and HeLa cells. **(C)** Levels of RIPK1- and RIPK3-specific mRNA in C4-I and HeLa cells were quantified using real-time PCR in relation to RPL13A. mRNA expression in C4-I cells was set at 100%. The mean values ± SD from *n* = 3 experiments performed in duplicate are shown. **(D)** Whole cell extracts of C4-I and HeLa cells were analyzed for RIPK1 and RIPK3 expression in Western blot using RIPK1-specific (left panels) or RIPK3-specific antibodies (right panels). Equal loading was controlled using a β-actin-specific monoclonal antibody. The results from one out of two independent experiments are shown.

To investigate further whether PolyIC induced features of necrosis in C4-I cells, we examined propidium iodide (PI) staining and lactate dehydrogenase (LDH) release. In strong contrast to HeLa cells, almost all C4-I cells incorporated PI after PolyIC stimulation (Figure 2A). Furthermore, PolyIC activation of C4-I led to an 80% release of LDH (Figure 2B). HeLa cells exhibited only a weak release of LDH (7%), which was completely abrogated in cell cultures that contained Z-VAD, indicating necrosis secondary to apoptosis in these cells (data not shown). These results suggested a form of cell death in C4-I cells that is characterized by caspase-3 activation (a typical feature of apoptosis), as well as regulated necrosis (also known as necroptosis).

### RIPK3, but not RIPK1, is differentially expressed in C4-I and HeLa cells

We analyzed the expression and impact of the receptor-interacting protein kinases RIPK1 and RIPK3, two intracellular factors that have been implicated in both necroptotic cell death and apoptosis depending on the biochemical context [38, 40, 41]. Quantitative RT-PCR demonstrated that RIPK1 was expressed equally in both cancer cell lines at the mRNA and protein levels (Figure 2C, 2D, left panels). In contrast, there was a huge difference between the cell lines regarding RIPK3 mRNA and protein expression. RIPK3 was only detected in C4-I cells, and not in HeLa cells (Figure 2C, 2D, right panels).

### RIPK3, but not RIPK1, is necessary for PolyIC-induced necroptotic cell death in C4-I cells and subsequent DC activation

**Figure 3:**
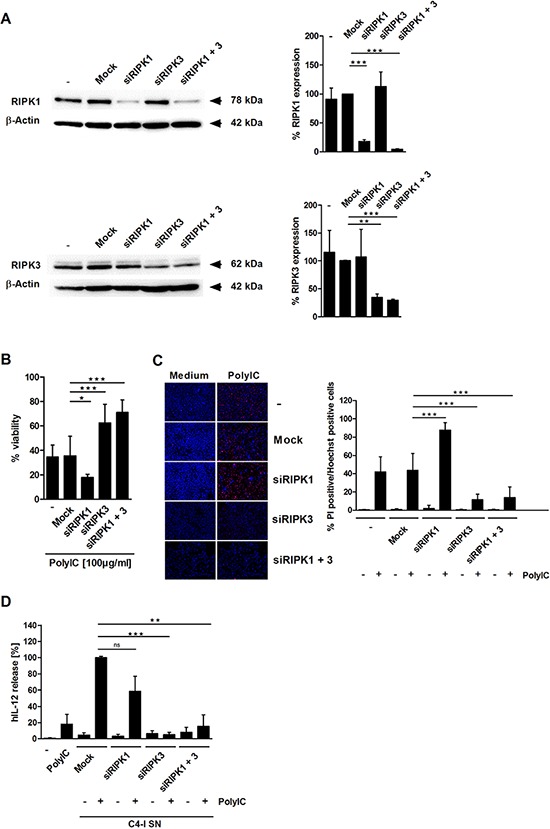
RIPK3 expression is required for PolyIC-induced cell death in C4-I cells **(A)** C4-I cells were transfected with specific siRNAs against RIPK1, RIPK3, both RIP kinases, or mock siRNA as a control. Whole cell extracts were analyzed for RIPK1 (upper panel) and RIPK3 (lower panel) expression in Western blot. Equal loading was controlled using a β-actin-specific monoclonal antibody. The diagram summarizes the results from *n* = 2 independent experiments. **(B–D)** C4-I cells were transfected as in (A). After 24 h, cells were stimulated with PolyIC. **(B)** Cell viability was assessed 24 h later using the neutral red uptake method, and the viability of mock transfected cells without PolyIC stimulation was set at 100%. The mean values ± SD from *n* = 3 experiments performed in triplicate are shown. **(C)** Transfected cells were stained with PI and Hoechst. Cells were analyzed using fluorescence microscopy at 200× magnification (left panel). Eight randomized pictures from *n* = 3 experiments performed in duplicate were taken per each condition and quantified for PI-positive cells (right panel). **(D)** Knockdown of RIPK3 in PolyIC-stimulated C4-I cells abolishes IL-12 induction in DC. DC were incubated with medium, PolyIC, or supernatants from medium- or PolyIC-treated C4-I cells transfected with specific siRNAs against RIPK1, RIPK3, both RIP kinases, or mock siRNA as a control. DC supernatants were analyzed for IL-12 expression. The mean values ± SD from *n* = 3 experiments performed in triplicate are shown.

We performed knockdown experiments to test the respective impacts of RIPK1 and RIPK3 on PolyIC-induced responses in C4-I cells. RIPK1 and RIPK3 siRNAs each specifically knocked down their respective RNA targets (Figure 3A). In both cases, the individual knockdown efficiencies exceeded 80% and transfection of each siRNA had no negative influence. Notably, only RIPK3-specific siRNA significantly reduced cell death induction in PolyIC-stimulated C4-I cells, as measured by neutral red uptake (Figure 3B) and PI staining (Figure 3C). This result was not altered by the simultaneous transfection of RIPK1 siRNA. In contrast, knockdown of RIPK1 alone even increased cell death (Figure 3B, 3C).

Next, we investigated whether the expression of either RIP kinase in C4-I cells affects their capacity to stimulate DC. Supernatants from siRNA-transfected and PolyIC-stimulated (or unstimulated) C4-I cells were collected and used in the DC activation assay (Figure 3D). Again, PolyIC-treated, mock-transfected cancer cells had a stronger effect on IL-12 production than direct PolyIC stimulation of the DC. Knockdown of RIPK1 in C4-I cells did not significantly alter IL-12 production in DC. Most importantly, in DC stimulated with conditioned media from C4-I cells in which RIPK3 was knocked-down, IL-12 production was potently and significantly suppressed (Figure 3D). These data demonstrated that RIPK3 is the key regulator of PolyIC-induced necroptosis in C4-I cells and is necessary for the enhancement of IL-12 production in DC.

### RIPK3 expression pattern in cervical cancers *in vivo*

**Figure 4:**
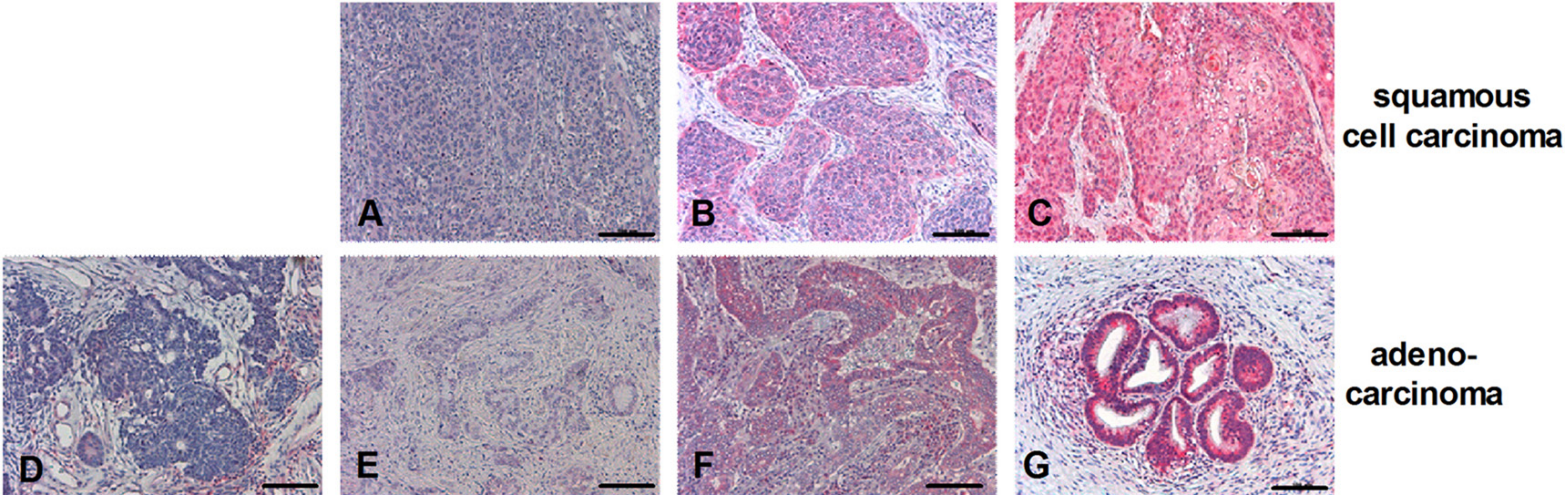
Expression patterns of RIPK3 in SCC or adenocarcinoma Paraffin-embedded biopsies of human SCC **(A–C)** or adenocarcinoma **(D–G)** were immunohistochemically stained with anti-RIPK3 antibody (red color; all 200 ×). Scale bars: 100 μm. RIPK3 staining intensities in cancer cells were classified according to IRS Score: 0–2 = negative **(D)**; 3–4 = positive, weak expression **(A, E)**; 6–8 = positive, moderate expression **(B, F)**; 9–12 = positive, strong expression **(C, G)**.

Because C4-I cells are derived from a cervical squamous cell carcinoma (SCC) and HeLa is an adenocarcinoma cell line, we were interested in the *in vivo* expression patterns of RIPK3 in both human cervical cancer entities. All cervical cancer specimens were HPV-typed as indicated in Table 1. Applying the immunoreactive score (IRS score) (Supplementary Table S1), RIPK3 expression was judged positive in 16/16 human cervical SCCs. Expression levels ranged from weak to strong and did not correlate with the HPV type (Figure 4A–4C and Table 1). Cervical adenocarcinomas displayed broader inter-individual and intra-tumoral heterogeneity with respect to RIPK3 expression (Figure 4F, 4G). While 2/10 cancers displayed very strong expression, 5/10 cancers were judged negative according to the IRS score. Interestingly, 3 of 5 RIPK3-negative adenocarcinomas were also HPV-negative (Figure 4D–4G and Table 1).

**Table 1:** RIPK3 expression in SCC and adenocarcinoma according to the IRS Score and HPV genotyping of the tissue specimens

Immunoreactive Score (IRS)	SCC Score (Frequency %)	HPV type			Adenocarcinoma Score (Frequency %)	HPV type			
		16	18	33		neg.	16	18	45
0–2 = negative	-				5 (50%)	3	1		1
3–4 = weak	9 (56.3%)	7	1	1	1 (10%)			1	
6–8 = moderate	4 (25.0%)	3	1		2 (20%)		1		1
9–12 = strong	3 (18.7)	3			2 (20%)	1		1	

### HMGB1 is released from PolyIC-stimulated C4-I cells, but does not enhance IL-12 production in DC

**Figure 5:**
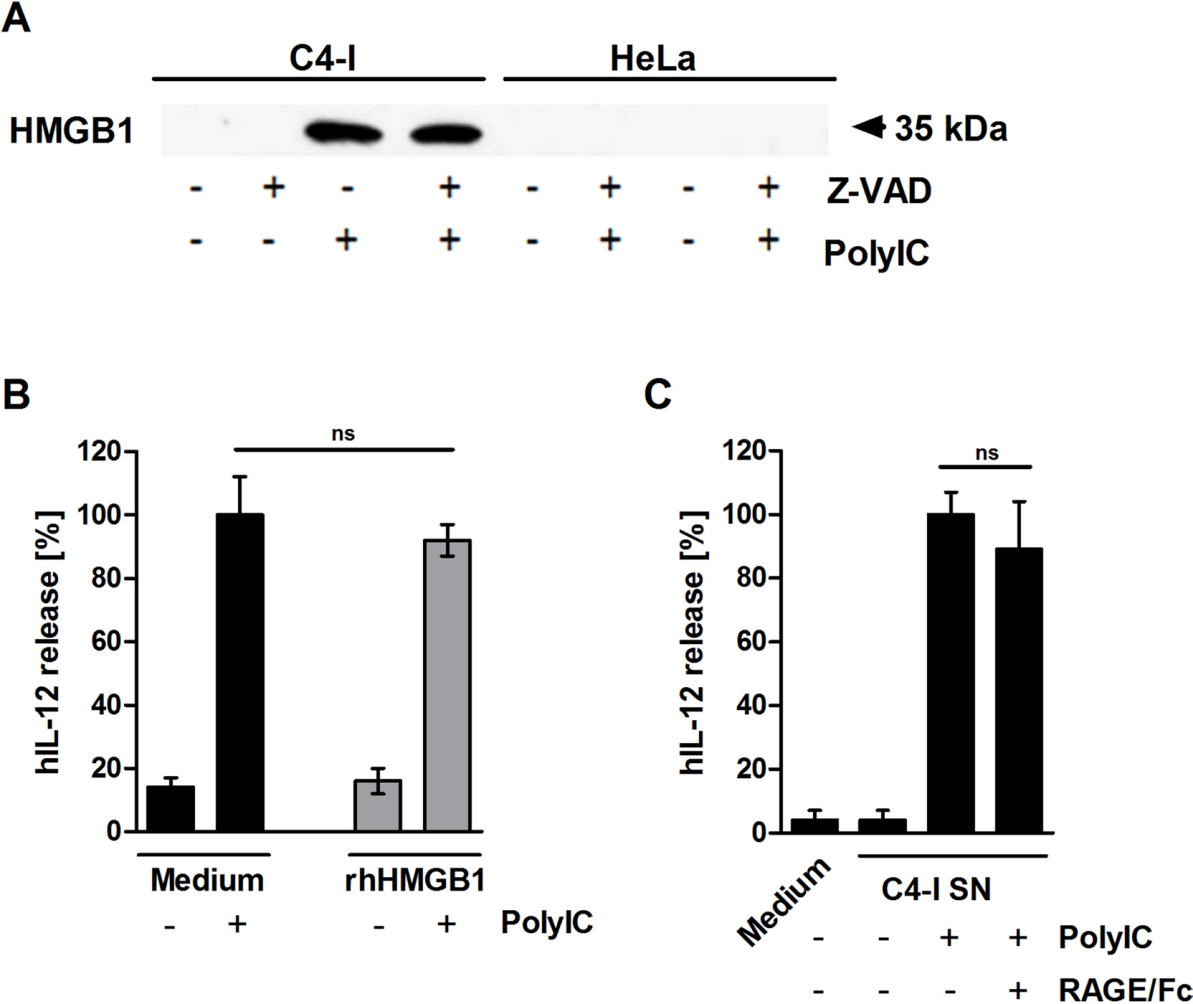
HMGB1 is released from PolyIC-stimulated C4-I cells **(A)** C4-I and HeLa cells were pre-incubated with Z-VAD for 30 min and stimulated with medium or PolyIC for 24 h. Equal amounts of supernatant were analyzed for HMGB1 release in Western blot using HMGB1-specific antibodies. **(B)** HMGB1 does not enhance PolyIC-induced IL-12 expression. C4-I cells were stimulated with medium or PolyIC in the absence (black bars) or presence (grey bars) of rhHMGB1. PolyIC-induced IL-12 expression in DC was set at 100%. The mean values ± SD from *n* = 2 experiments performed in duplicate are shown. **(C)** DC were stimulated with medium or supernatant from PolyIC-treated C4-I cells in the absence or presence of RAGE/Fc. The IL-12 expression induced by supernatant from PolyIC-treated C4-I cells was set at 100%. The mean values ± SD of *n* = 3 experiments performed in duplicate are shown.

We were interested in determining which factor released during PolyIC-mediated necroptosis was responsible for the enhancement of DC activation. HMGB1, an alarmin with immunostimulatory capacity that is passively released during necrosis [42], was found in supernatants from PolyIC-stimulated C4-I cells, but not in supernatants from HeLa cells. Z-VAD did not affect HMGB1 release (Figure 5A). A loading control for this experiment is shown in Supplementary Figure S2. Recombinant HMGB1 did not directly activate DC or enhance the effect of PolyIC (Figure 5B). Naturally expressed HMGB1 may be post-translationally modified, and may differ from bacterially expressed HMGB1 with respect to functional activity. Therefore, supernatants from PolyIC-activated C4-I cells were neutralized using the soluble receptor construct RAGE/Fc [43]. RAGE/Fc did not significantly interfere with the IL-12 production induced by supernatants from PolyIC-stimulated C4-I cells (Figure 5C). These data indicated that HMGB1 was released during PolyIC-mediated necroptosis, but was not responsible for enhanced IL-12 production in DC.

### RIPK3-dependent IL-1α release from PolyIC-stimulated C4-I cells enhances IL-12 production in DC

**Figure 6:**
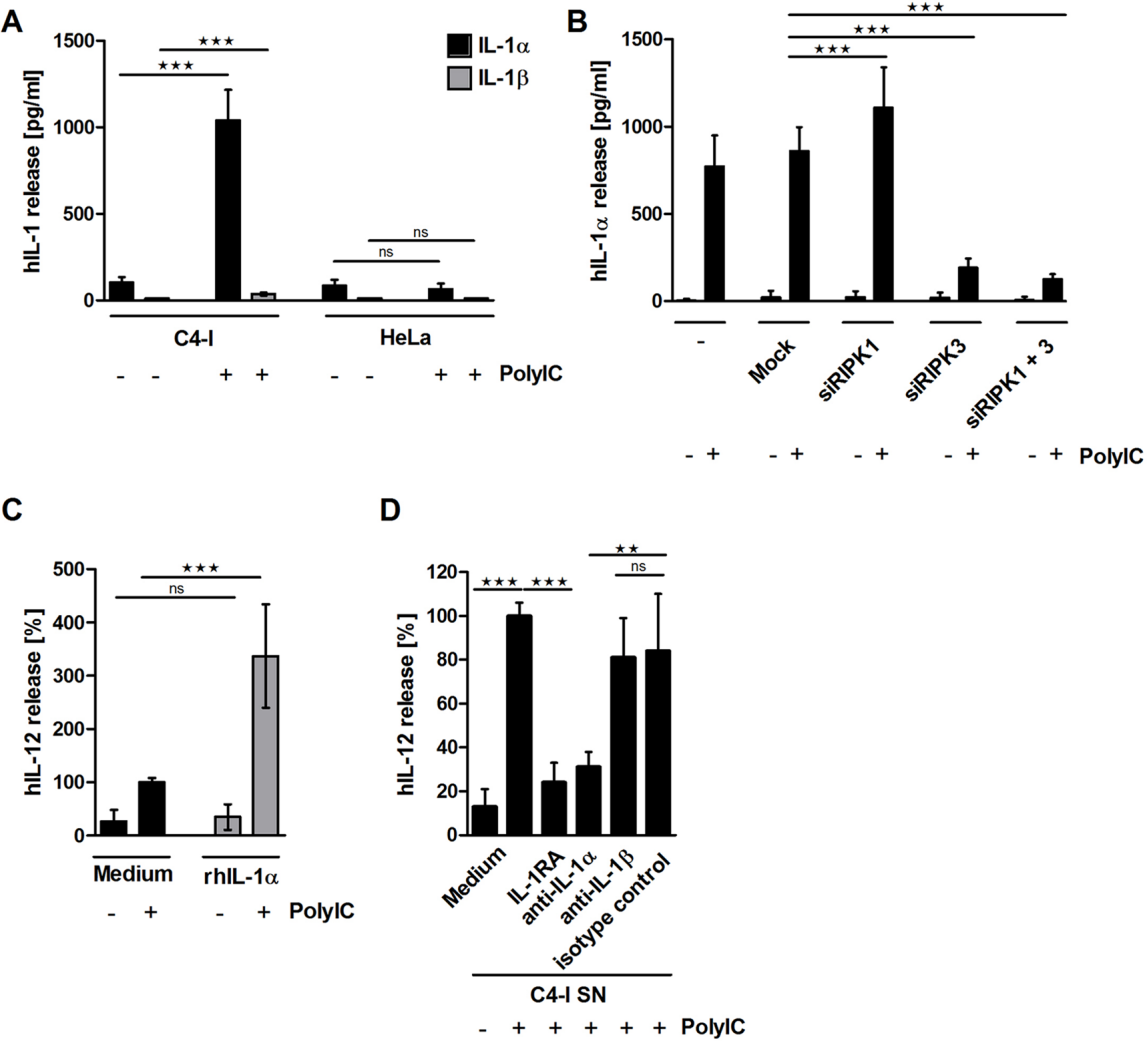
**(A) PolyIC-stimulated C4-I cells release IL-1α, but not IL-1β.** C4-I and HeLa cells were stimulated with medium or PolyIC for 24 h. Supernatants were analyzed for IL-1α (black bars) and IL-1β (grey bars) content. The mean values ± SD from *n* = 3 experiments performed in triplicate are shown. **(B)** RIPK3 expression is required for IL-1α release from PolyIC-treated C4-I cells. C4-I cells were transfected with specific siRNAs against RIPK1, RIPK3, both RIP kinases, or mock siRNA as a control. After 24 h, cells were stimulated with PolyIC. Supernatants were analyzed for IL-1α expression using ELISA. The mean values ± SD from *n* = 3 performed in duplicate are shown. **(C)** IL-1α release from PolyIC-treated C4-I cells is necessary to enhance DC activation. DC were stimulated with medium or rhIL-1α in the absence or presence of PolyIC. PolyIC-induced IL-12 expression was set at 100%. The mean values ± SD from *n* = 3 experiments performed in duplicate are shown. **(D)** DC were stimulated with supernatant from PolyIC-treated C4-I cells in the presence of IL-1RA, neutralizing antibodies against IL-1α or IL-1β, or the respective isotype control. The IL-12 expression induced by supernatant from PolyIC-treated C4-I cells was set at 100%. The mean values ± SD from *n* = 4 experiments performed in duplicate are shown.

IL-1α was regarded as another interesting candidate in the search for other immunostimulatory factors released from PolyIC-stimulated necroptotic C4-I cells. Keratinocytes constitutively express preformed IL-1α precursor (summarized by Dinarello in 2011 [44]) and, unlike other inflammatory cytokines, HPV oncoproteins apparently do not interfere with its expression [31]. In fact, PolyIC-stimulated C4-I cells, but not HeLa cells, released high amounts of IL-1α, while IL-1β was barely detectable in supernatant from either of these cell lines (Figure 6A).

Next, we investigated the impact of the RIP kinases on IL-1α release in PolyIC-stimulated necroptotic C4-I cells. Knockdown of RIPK3, but not RIPK1, significantly suppressed PolyIC-induced IL-1α release, which corresponded well with the cell death experiments (Figure 6B).

We were also interested in whether recombinant human (rh)IL-1α was able to enhance IL-12 production in DC in the absence or presence of PolyIC. We found that rhIL-1α did not induce IL-12 release in DC when applied alone; however, it strongly enhanced PolyIC-mediated IL-12 release in DC (Figure 6C). We performed neutralization experiments in order to analyze the impact of endogenous IL-1α released from PolyIC-stimulated C4-I cells on DC activation. Either IL-1 receptor antagonist (IL-1RA), or specific IL-1α- or IL-1β-neutralizing antibodies were added to supernatant from the PolyIC-stimulated C4-I cells. IL-1RA suppressed IL-12 production by 72%. Similarly, neutralizing antibodies against IL-1α decreased IL-12 production by approximately 75% (Figure 6D). In contrast, IL-1β-neutralizing antibodies did not significantly affect IL-12 production by DC.

These data clearly show that during the PolyIC-induced cell death of cervical cancer cells, RIPK3 expression is crucial for the release of IL-1α, which links cancer cell necroptosis with potent DC activation.

## DISCUSSION

There is a strong need for novel therapeutic strategies against cervical cancer. The dsRNA PolyIC has been proposed as an adjuvant to enhance immunity against cancer. However, it was unclear whether and how PolyIC-treated HPV-positive cells might be able to activate professional antigen-presenting cells, since they generally express only low amounts of inflammatory cytokines.

Here we show that PolyIC-stimulated cervical cancer cells can induce 10-fold greater IL-12 production in DC than direct PolyIC stimulation of DC. This immunostimulatory cytokine is critical for effective anti-tumor responses [12, 13, 45]. This study has clarified the molecular mechanism underlying this strong enhancement of DC activation. We demonstrated that the release of the alarmin IL-1α during PolyIC-induced necroptosis in cervical cancer cells was necessary for an effective IL-12 response. Necroptosis, IL-1α release, and enhancement of DC activation were dependent on the expression of RIPK3 in cancer cells. Our *in situ* analyses of human cervical cancer specimens revealed heterogeneous RIPK3 expression patterns in cervical SCCs and adenocarcinomas. This study has identified a novel mechanism that explains how PolyIC-treatment of cervical cancer cells results in effective DC activation. These findings have important implications for future personalized adjuvant immunotherapy strategies.

A striking finding of our study was that PolyIC-mediated IL-1α release from cervical cancer cells is required in order to enhance IL-12 production by DC. Given the results of other studies involving PolyIC-treated normal keratinocytes and different cancer types, one would expect that IFNβ and/or TNFα are needed for DC activation [20, 22, 46]. However, we were unable to detect the release of these cytokines in supernatant from cervical cancer cells after PolyIC stimulation. One characteristic of cervical cancer is infection with oncogenic HPV. Earlier studies have clearly demonstrated that mucosal high-risk HPV oncoproteins strongly suppress both constitutive and PolyIC-induced inflammatory cytokines in keratinocytes [24–28]. Inflammatory cytokine production is also low to absent in HPV-transformed and cervical cancer cells [29, 30, 32, 34]. IL-1β expression in particular declines gradually during cervical carcinogenesis [25]. This is in strong contrast to IL-1α, which has been detected in HPV-infected epithelia during all stages of malignancy [31]. Thus, IL-1α seems to be an exception from the rule of cytokine suppression during cervical carcinogenesis, similar to IL-6 [32].

In fact, neither neutralization of the low amounts of IL-1β produced after PolyIC-treatment of the cancer cells nor caspase-1 or pan-caspase inhibitors, both of which have the potential to interfere with IL-1β processing and activation [47, 48], significantly inhibited DC activation. Our study provides clear evidence that in the context of PolyIC stimulation, IL-1α release from cervical cancer cells can potentiate IL-12 production by DC. Aside from IL-1α, another alarmin, HMGB1, was also strongly released during PolyIC-induced necroptosis. Although HMGB1 had no effect on IL-12 induction, this does not exclude a potential role for HMGB1 in the activation of other immune functions [49].

We unraveled the kinase RIPK3 as the critical determinant for IL-1α release and for the DC-stimulatory capacity of PolyIC-treated cervical cancer cells. The crucial role of RIPK3 was identified by comparing two cervical cell lines with differential DC-stimulatory capacities, the cervical SCC cell line C4-I expressing RIPK3 (as shown in this study) and the HeLa cervical adenocarcinoma cell line, which is known to be devoid of RIPK3 expression [50]. RIPK3 expression was also observed in the HPV16-positive cell line HPKIA demonstrating that it was not restricted to HPV18-transformed cells (Supplementary Figure S3A). Moreover, all HPV16-positive cervical SCC and 1 out of 2 HPV16-positive adenocarcinomas expressed RIPK3 *in situ* (Table 1). Similar to C4-I cells, HPKIA potently enhanced the stimulatory effects of PolyIC on DC (Supplementary Figure S3B). RIPK3 knockdown in C4-I cells not only suppressed PolyIC-induced cell death and IL-1α release in neoplastic cells, but also suppressed subsequent IL-12 induction in DC. Whether or not cancer necroptosis was essential for the release of IL-1α is difficult to judge [51], because both cell death and IL-1α release were dependent on regulation by RIPK3.

In contrast to RIPK3, RIPK1 expression was dispensable for IL-1α release. This finding was most striking because IL-1α production is regulated by RIPK1 independently of RIPK3 in murine hematopoietic cells [52]. Although RIPK3 contributes to the generation of active IL-1β in other cell types [53, 54], the release of IL-1β from PolyIC-stimulated cervical cancer cells was negligible and did not contribute significantly to DC stimulation. However, the low IL-1β expression in C4-I and HeLa cells might be explained by the IL-1β-suppressive effects of HPV [27].

Our *in vivo* analyses in cervical cancers revealed that RIPK3 is expressed in all SCCs investigated thus far, at varying levels ranging from weak to strong. Notably, we found an even more heterogeneous RIPK3 expression pattern in cervical adenocarcinomas; we observed highly positive examples, as well as cancers with a high percentage of cells that exhibited no RIPK3 expression, which were therefore judged negative using the IRS score. Thus, one may speculate that HeLa cells were derived from a RIPK3-negative adenocarcinoma or a negative part of a tumor with heterogeneous RIPK3 expression.

There is ample evidence that HPV oncoproteins not only interfere with inflammatory responses, but also suppress apoptosis at various levels [7]. This includes the degradation of p53 [6], as well as Fas-associated death domain (FADD) and procaspase-8 [55, 56]. This study demonstrates for the first time that potent induction of immunostimulatory necroptotic cell death is feasible in cervical cancer cells and requires the expression of RIPK3. This finding is consistent with previous studies that characterized RIPK3 as the key regulator of TNFα-induced programmed necrosis [57–60]. The fact that FADD, a target of HPV, is a negative regulator of RIPK3-mediated necrosis [61, 62] may explain the strong necroptotic response in HPV-positive cancer cells that express RIPK3.

While other studies have also implicated RIPK1 in necroptotic cell death [63], RIPK1 knockdown did not interfere with PolyIC-induced necroptosis. Instead, it led to enhanced cell death. These unexpected data corresponded very well with recently published observations that RIPK1 can suppress spontaneous RIPK3 activation and necroptosis *in vitro* and act as an inhibitor of epithelial apoptosis and necrosis in different murine models ensuring tissue homeostasis [64–67]. It also emphasizes the potentially broader relevance of our finding for human cancer cells.

In summary, our study has identified a novel RIPK3- and IL-1α-dependent mechanism that links dsRNA treatment of cervical cancer cells with DC activation. These findings have important implications for future treatment options in cervical cancer patients. Our data suggest that the expression status of RIPK3 might critically influence the outcome of PolyIC-based adjuvant immunotherapeutic approaches in cervical (and potentially other) cancers and should therefore be assessed prior to immunotherapy. Furthermore, we speculate that other dsRNA mimics or oncolytic RNA viruses that employ molecular mechanisms similar to that of PolyIC might also lead to enhanced immune activation in RIPK3-expressing cancers.

## MATERIALS AND METHODS

### Immunohistochemical analysis and HPV-genotyping of cervical cancer specimens

Paraffin-embedded, formalin-fixed tissue samples from 26 patients with cancerous lesions of the cervix uteri were taken from the local pathology archive at Saarland University, Germany. This retrospective study has been conducted according to Declaration of Helsinki principles, and the local Ethics Committee of the Saarland University approved the protocols. Ten-micrometer thick sections were investigated by gp5+/6+ PCR as described in [68] and by subsequent sequencing of the PCR product and/or genotyped by the INNO-LiPA HPV Genotyping assay (Fujirebio Europe, Gent, Belgium) according to the manufacturer's instructions. Two-micrometer thick sections were deparaffinized. Histological diagnosis of tissues stained with hematoxylin and eosin (H&E) was ascertained by expert pathologists (YJK or RMB). For immunohistochemistry, rabbit anti-RIPK3 antibody (Thermo Scientific, Bonn, Germany) and the ImmPRESS Detection Kit (Vector Laboratories, Burlingame, USA) were used. Slides were evaluated with a DMI 6000B microscope (Leica, Wetzlar, Germany) and Microsoft Image Composite Editor program using standardized settings. RIPK3 staining intensity was classified using the Immunoreactive Score (IRS) according to Remmele & Stegner (Supplementary Table S1). Biopsies were evaluated as negative (IRS 0–2) or positive (IRS 3–12; 3–4 = weak, 6–8 = moderate, and 9–12 = strong expression).

### Cervical cancer cell culture

HPV18-positive cervical carcinoma cell lines HeLa (ATCC CCL-2) and C4-I (TCC CRL-1594), obtained from M. von Knebel–Doeberitz, were authenticated by qRT-PCR for HPV16 or HPV18 E6 and E7 expression, as well as by Multiplexion (DKFZ, Germany) in April 2013, and cultured as previously described [33]. To obtain conditioned media, cells were cultured at a density of 1 × 10^6^ cells/ml. After 24 h, cells were stimulated with 100 μg/ml PolyIC (Amersham Biosciences, Freiburg) in RPMI (Gibco, Karlsruhe, Germany) supplemented as described [33] for an additional 24 h. Supernatants were harvested and stored at −20°C. In some experiments, cells were pre-incubated with 5 μM Z-VAD or 5 μM Y-VAD (Axxora GmbH, Lörrach, Germany) for 30 min. Alternatively, neutralizing anti-IL-1α, anti-IL-1β, isotype-control antibody (2 μg/ml), or a chimeric receptor consisting of the Fc region of IgG_1_ fused to the extracellular domain of the RAGE receptor (10 μg/ml, all from R&D Systems, Minneapolis, USA) [43] were added to the conditioned media.

### Monocyte isolation, DC culture, and IL-12 induction experiment

Peripheral blood mononuclear cells were isolated from buffy coats or whole blood from healthy donors (written informed consent was obtained and the protocol was approved by the local Ethics Committee) as previously described [34, 69]. For DC differentiation, monocytes were cultured at a density of 1 × 10^6^ cells/ml for 6 days in six-well plates as indicated (Renner GmbH, Dannstadt, Germany) in RPMI medium supplemented with GM-CSF (100 ng/ml Leukine; Berlex, Montville, NJ) and IL-4 (5 ng/ml; PeproTech, Rocky Hill, CT) [33]. On day 6, immature DC were stimulated with medium, 100 μg/ml PolyIC, or supernatant from PolyIC-treated cervical cancer cells for 8 h, followed by three washing steps. In a 24-well plate, 1.5 × 10^5^ DC/0.5 ml/well were stimulated with 4.5 × 10^5^ paraformaldehyde-fixed BHK-CD40L cells, which were prepared as described earlier [70]. In some experiments they were stimulated with recombinant human HMGB1 (rhHMGB1, 400 pg/ml, R&D Systems) or rhIL-1α (5 ng/ml, PeproTech).

### Transfection of siRNAs

In a 6-well plate, 30 pmol of indicated siRNAs per 2.5 × 10^5^ C4-I cells/well (ON-TARGETplus Non-targeting siRNA #2, ON-TARGET smartpool for RIPK1 or RIPK3, all from Thermo Scientific, Bonn, Germany) were transfected with Lipofectamine RNAiMax (Invitrogen) according to the manufacturer's guidelines. Forty-eight hours later, protein amounts were analyzed by Western blot analysis or used in assays.

### Cellular lysates and western blot analysis

Cells were seeded at a density of 1 × 10^6^ cells/dish (dishes were 6 cm diameter). Twenty-four hours later, they were stimulated with medium or PolyIC for the indicated time intervals. Cells were resuspended in sample buffer (62.5 mM Tris-HCl pH 6.8, 4% SDS, 20% glycerol, 100 mM DTT), and equal amounts of protein were analyzed using antibodies directed against RIPK3 (Thermo Scientific), RIPK1 (Cell Signaling Technologies, Frankfurt, Germany), caspase-3 (Cell Signaling Technologies), HMGB1 (R&D Systems), or β-actin (Sigma-Aldrich, Taufkirchen, Germany) as a loading control. Secondary antibodies (Sigma-Aldrich) and Supersignal West Dura Luminol reagent (Thermo Scientific) were used for standardized detection with a ChemiDoc XRS+ Molecular Imager. Quantification was performed using the Quantity One analysis software (both BioRad, Philadelphia, PA).

### Cell death assays

For each assay, 3 × 10^4^ cells/well in a 96-well plate were stimulated with medium or serial dilutions of PolyIC for 24 h. Cell viability was assessed by the neutral red up-take method as described [70], by crystal violet staining, or using the LDH-Cytotoxicity Detection Kit (Roche, Mannheim, Germany) according to manufacturer's protocol. In the latter protocol, cells treated with 2% Triton X-100 (Serva, Heidelberg, Germany) were used as positive control. For Hoechst/PI assays, 0.5 × 10^6^ cells/well in a 6-well plate were sequentially stained with 10 μM H33342 (Sigma-Aldrich, Taufkirchen, Germany) and 5 μg/ml PI (Sigma-Aldrich). Cells were analyzed using a DMI 6000B fluorescence microscope (Leica, Wetzlar, Germany) at 200× magnification; eight images were taken per condition and analyzed for Hoechst/PI-positive cells using the appropriate FW4000 software (Leica).

### Quantitative real-time PCR

RNA isolation, cDNA synthesis, real-time PCR, and normalization to RPL13A or glyceraldehyde 3-phosphate dehydrogenase (GAPDH) were performed as previously described [33, 71]. The 83-bp fragment of RIPK3 was detected with primers 5′-GCCTCCACAGCCAG TGAC-3′ and 5′-TCGGTTGGCAACTCAACTT-3′ and probe no. 11; the 94-bp fragment of RIPK1 was detected with primers 5′-AGGAAATACACCCACCATGC-3′ and 5′-TCCAATCTGAATGCCAGTACTATT-3′ and probe no. 17.

### ELISA

IL-1α, IL-1β, and IL-12 concentrations were determined with DuoSet (R&D Systems), TNFα concentration was determined with the OptEIASet (BD Biosciences, Heidelberg, Germany), and IFNβ concentration was determined with polyclonal anti-IFNβ antibody (Ab) AB1443 and monoclonal Ab A1 (Millipore, Schwalbach, Germany) according to the suppliers’ instructions. Detection limits were 7.8 pg/ml (IL-1α, TNFα, IL-12), 3.9 pg/ml (IL-1β), and 19.5 pg/ml (IFNβ).

### Statistical analysis

To evaluate the statistical differences between analyzed groups, a two-sided *t*-test was applied. Statistical significance is indicated by asterisks (*p < 0.05, **p < 0.01, ***p < 0.001).

## SUPPLEMENTARY FIGURES AND TABLE


