# Correction: RIPK3 expression in cervical cancer cells is required for PolyIC-induced necroptosis, IL-1α release, and efficient paracrine dendritic cell activation

**DOI:** 10.18632/oncotarget.27066

**Published:** 2019-07-09

**Authors:** Susanne V. Schmidt, Stefanie Seibert, Barbara Walch-Rückheim, Benjamin Vicinus, Eva-Maria Kamionka, Jennifer Pahne-Zeppenfeld, Erich-Franz Solomayer, Yoo-Jin Kim, Rainer M. Bohle, Sigrun Smola

**Affiliations:** ^1^ Center for Molecular Medicine Cologne and Institute of Virology, University of Cologne, Germany; ^2^ Institute of Virology, Saarland University, Homburg/Saar, Germany; ^3^ Department of Gynecology and Obstetrics, Saarland University, Homburg/Saar, Germany; ^4^ Department of Pathology, Saarland University, Homburg/Saar, Germany

**This article has been corrected:** In Figure 3C, cells were stained with propidium iodide (PI) and nuclei were counterstained with “Hoechst” stain. Cells were then analyzed using fluorescence microscopy at 200x magnification, as shown in the left panel. However, in the lowest left picture of Figure 3C (left panel, condition “Medium; siRIPK1+3”), the correct PI stain was mistakenly merged with a false Hoechst counterstain picture. Erroneously, the Hoechst stain photograph representing “PolyIC; siRIPK1+3” was used. Thus, for both conditions (“Medium” and “PolyIC”), the pictures look identical for blue nuclear staining (Hoechst) but different for red staining (PI). The corrected Figure 3 is shown below. The authors declare that these corrections do not change the results or conclusions of this paper.

Original article: Oncotarget. 2015; 6:8635–8647. 8635-8647 . https://doi.org/10.18632/oncotarget.3249

**Figure 3:**
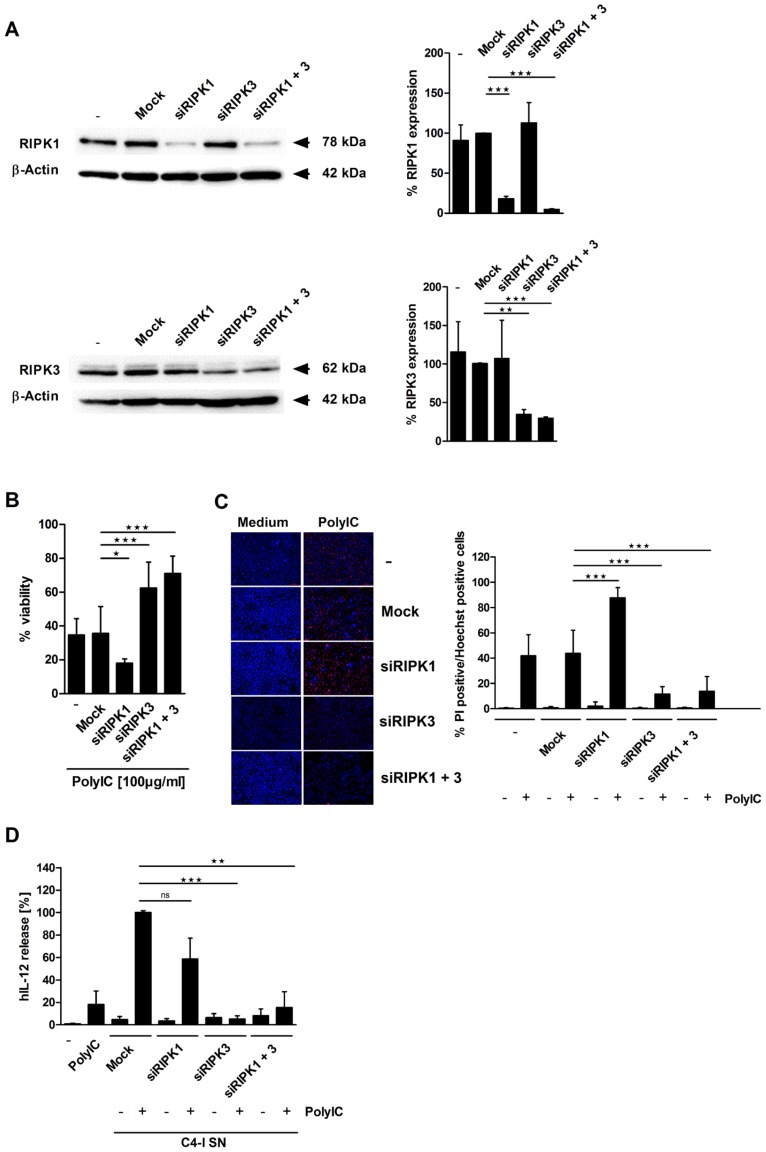
RIPK3 expression is required for PolyIC-induced cell death in C4-I cells. (**A**) C4-I cells were transfected with specific siRNAs against RIPK1, RIPK3, both RIP kinases, or mock siRNA as a control. Whole cell extracts were analyzed for RIPK1 (upper panel) and RIPK3 (lower panel) expression in Western blot. Equal loading was controlled using a β-actin-specific monoclonal antibody. The diagram summarizes the results from *n* = 2 independent experiments. (**B**–**D**) C4-I cells were transfected as in (A). After 24 h, cells were stimulated with PolyIC. (**B**) Cell viability was assessed 24 h later using the neutral red uptake method, and the viability of mock transfected cells without PolyIC stimulation was set at 100%. The mean values ± SD from *n* = 3 experiments performed in triplicate are shown. (**C**) Transfected cells were stained with PI and Hoechst. Cells were analyzed using fluorescence microscopy at 200× magnification (left panel). Eight randomized pictures from *n* = 3 experiments performed in duplicate were taken per each condition and quantified for PI-positive cells (right panel). (**D**) Knockdown of RIPK3 in PolyIC-stimulated C4-I cells abolishes IL-12 induction in DC. DC were incubated with medium, PolyIC, or supernatants from medium- or PolyIC-treated C4-I cells transfected with specific siRNAs against RIPK1, RIPK3, both RIP kinases, or mock siRNA as a control. DC supernatants were analyzed for IL-12 expression. The mean values ± SD from *n* = 3 experiments performed in triplicate are shown.

