# Supplementary material for: RIPK3 expression in cervical cancer cells is required for PolyIC-induced necroptosis, IL-1α release, and efficient paracrine dendritic cell activation
Source: Oncotarget. 2015 Mar 20;6(11):8635–47. doi: 10.18632/oncotarget.3249 (PMC4496172; doi:10.18632/oncotarget.3249)
Supplement: Supplementary file 1 [file oncotarget-06-8635-s001.pdf]

SUPPLEMENTARY FIGURES AND TABLE

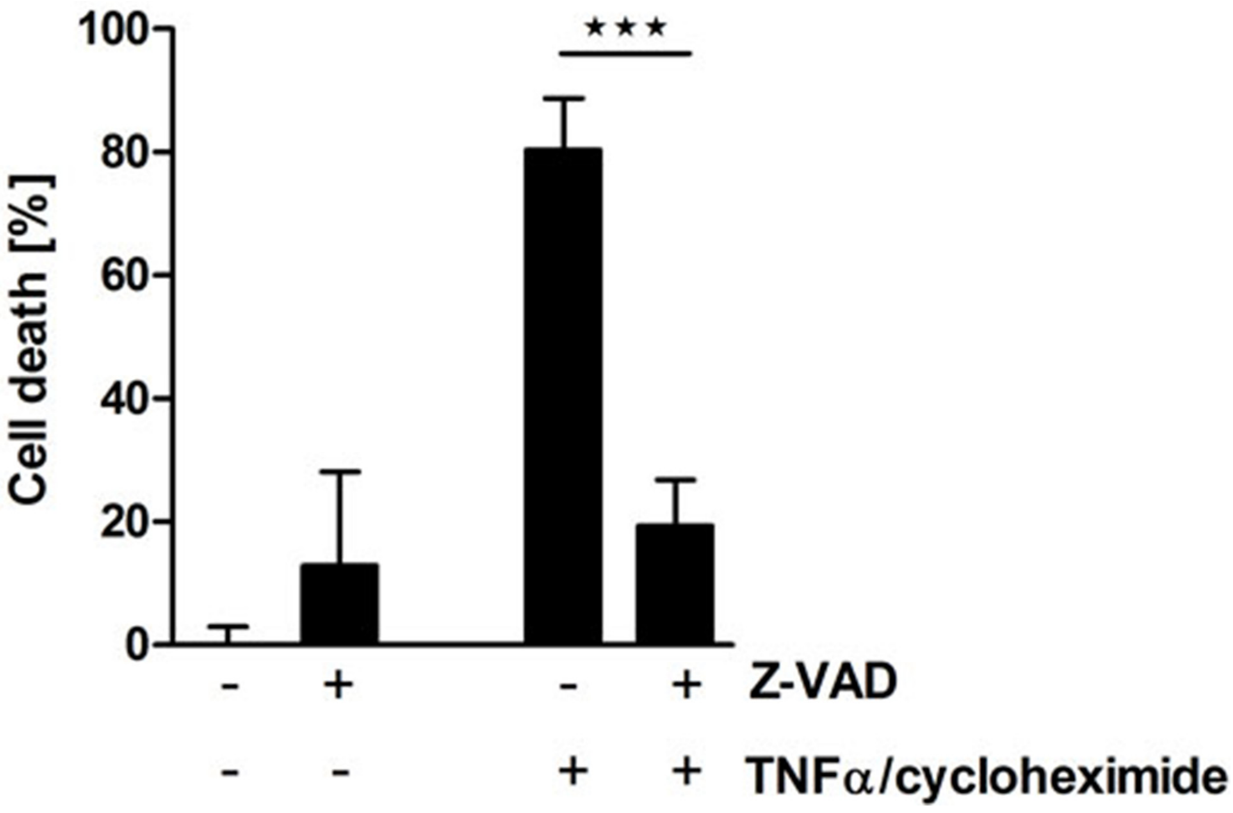

Supplementary Figure S1: HeLa cells were incubated with Z-VAD for 30 min, stimulated with 200 U/ml TNFα and 50 µg/ml cycloheximide for 24 h, and assessed for cellular viability. Viability of medium-treated cells was set at 100%. The mean values ± SD from *n* = 2 experiments performed in duplicate are shown.

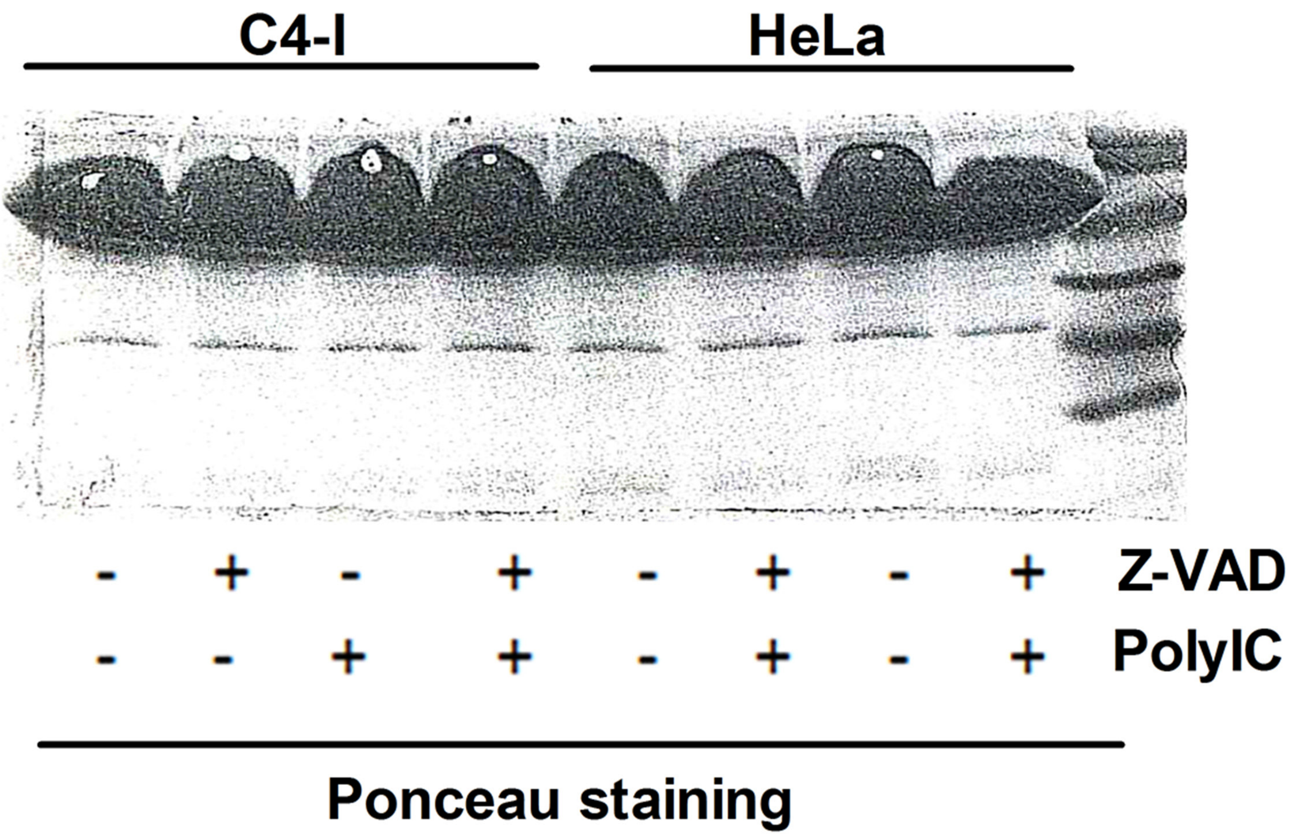

Supplementary Figure S2: C4-I and HeLa cells were pre-incubated with Z-VAD for 30 min and stimulated with medium or PolyIC for 24 h. Equal amounts of supernatant were analyzed in Western blot. Equal loading was controlled by Ponceau red staining.

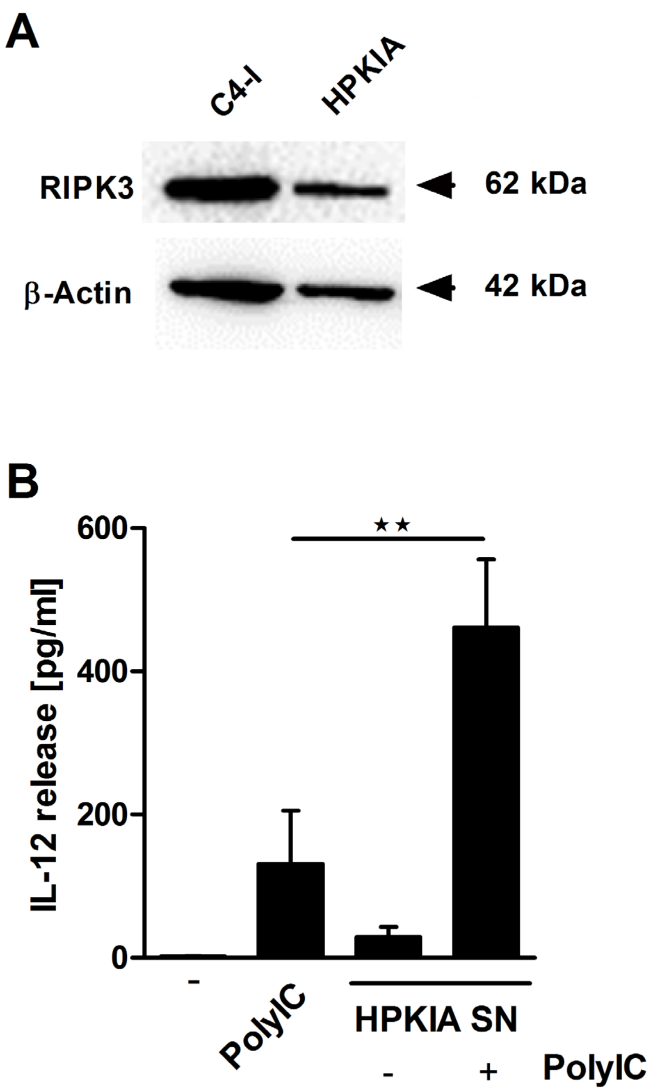

Supplementary Figure S3: (A) Whole cell extracts of C4-I and HPKIA cells were analyzed for RIPK3 expression in Western blot using RIPK3-specific antibodies. Equal loading was controlled using a β-actin-specific monoclonal antibody. The results from one out of two independent experiments are shown. **(B)** Supernatant from PolyIC-stimulated HPKIA cells enhances the IL-12 production of DC. DC were incubated with medium, PolyIC, or supernatants from HPKIA cells treated with medium or PolyIC. The resulting supernatants from stimulated DC were analyzed for IL-12 expression by ELISA. The mean values ± SD from $n = 3$ experiments performed in duplicate are shown.

Supplementary Table S1: Immunoreactive Score (IRS)

A (% of positive cells)	B (Staining intensity)	A × B = IRS
4 = > 80% positive cells		
3 = 51–80% of positive cells	3 = strong colour reaction	9–12 = strong
2 = 10–50% of positive cells	2 = moderate colour reaction	6–8 = moderate
1 = < 10% of positive cells	1 = weak colour reaction	3–4 = weak
0 = no positive cells	0 = no colour reaction	0–2 = negative
